# MicroRNA-mediated regulation of *KRAS* in cancer

**DOI:** 10.1186/s13045-014-0084-2

**Published:** 2014-11-30

**Authors:** Minlee Kim, Frank J Slack

**Affiliations:** Department of Molecular, Cellular and Developmental Biology, Yale University, PO Box 208103, New Haven, CT 06511 USA; Department of Pathology, Beth Israel Deaconess Medical Center, 330 Brookline Ave, Boston, MA 02215 USA

**Keywords:** microRNA (miRNA), *KRAS*, Cancer, Sequence variant, Single nucleotide polymorphism (SNP), *KRAS*-variant, rs61764370, rs712

## Abstract

While microRNAs (miRNAs) and the *KRAS* oncogene are known to be dysregulated in various cancers, little is known about the role of miRNAs in the regulation of *KRAS* in cancer. Here we review a selection of studies published in 2014 that have contributed to our understanding of the molecular mechanisms of *KRAS* regulation by miRNAs and the clinical relevance of sequence variants that may interfere with functional miRNA-mediated *KRAS* regulation.

## Background

Since their discovery about two decades ago, the profound role of microRNAs (miRNAs) in various aspects of cancer is being uncovered including in cancer therapy [1,2]. MiRNAs modulate a wide range of biological processes, such as cellular proliferation, differentiation and apoptosis, canonically by binding to the 3′ UTR of mRNAs by partial complementarity and inhibiting mRNA stability and translation. Since oncogenic *KRAS* is frequently found in many cancers, including colon, pancreatic, and lung cancer, and different cancer types and stages exhibit distinctive miRNA profiles, the regulation of *KRAS* by miRNAs has drawn attention in the field. The *KRAS* oncogene, which encodes a GTPase signaling protein, is a key driver of complex, multistep tumorigenesis, as alteration and activation of the gene and its pathway lead to acquisition of cancerous properties [3].

Here we review the studies published in 2014 that explored miRNA-mediated regulation of *KRAS* in different cancers. We briefly discuss the tumor-suppressive role of miRNAs that target and regulate *KRAS* and the regulation of those miRNAs (Table 1). In addition, the clinical potential of sequence variants in the 3′ UTR of *KRAS* (Table 2) as a cancer biomarker by altering the function of miRNAs is discussed.

**Table 1:** **MiRNAs that regulate** ***KRAS*** **cited in this Research Highlight**

**miRNA**	**Cancer type**	**Reference**
*let*-*7*	Lung cancer	[4]
miR-96	Pancreatic cancer	[5]
miR-30c	Hereditary breast cancer	[6]
miR-181a	Oral squamous cell carcinoma	[7]
miR-193b/365a	Cutaneous squamous cell carcinoma (cSCC)	[8]
miR-30b	Colorectal cancer (CRC)	[9]
miR-96	Pancreatic ductal adenocarcinoma (PDAC)	[10]
miR-134	Glioblastoma (GBM)	[11]

**Table 2:** **SNPs in the 3**′ **UTR of** ***KRAS*** **associated with cancer**

**SNP ID**	**Association with cancer**	**Reference**
rs61764370 (*KRAS*-variant)	Risk of non small-cell lung cancer, epithelial ovarian cancer, triple-negative breast cancer, colorectal cancer. Drug response in metastatic colorectal cancer	[12-16,18,19]
rs712	Risk of oral squamous cell carcinoma, gastric cancer, colorectal cancer, papillary thyroid cancer	[25-28]

### MiRNAs that target and regulate *KRAS* act as tumor suppressors

The seminal study by Johnson *et al*. identified the *let*-*7* family of miRNAs as the first tumor-suppressive miRNA known to target and regulate *KRAS* [4]. Subsequently, other tumor-suppressive miRNAs, including miR-96, miR-30c and miR-181a, have shown to regulate *KRAS* in various cancers [5-7]. More recently, Gastaldi *et al*. have utilized a large scale profiling technology, small RNA sequencing, to profile miRNAs in cutaneous squamous cell carcinomas (cSCCs) and identified the miR-193b/365a cluster as one of the most prominently down-regulated miRNAs in murine skin tumor progression [8]. Their role as a tumor suppressor was confirmed in both mouse and human epidermis, as these two miRNAs modulated cellular proliferation, migration and clonogenic potential. Functional assays that showed an inverse relationship between the miRNAs and KRAS protein levels validated that the two miRNAs functioned through targeting *KRAS*. Additionally, the effects of the miRNAs were recapitulated with *KRAS* knockdown in squamous carcinoma cells [8].

While several miRNA expression profiles report deregulation of numerous miRNAs in various cancers, only a few miRNAs have been characterized. Liao *et al*. further investigated the role of miR-30b, one of the known down-regulated miRNAs in colorectal cancer (CRC) [9]. The clinical relevance of miR-30b was shown in a cohort of 91 CRC cases, in which the level of miR-30b was correlated with poor progression and survival. Ectopic expression and inhibition of miR-30b affected cellular proliferation in CRC cell lines and tumor growth in a xenograft mouse model as miR-30b promotes G1 cell-cycle arrest and apoptosis. The effect of miR-30b in tumor growth was mediated through targeting many genes including *KRAS* [9].

### Regulation of tumor-suppressive miRNAs that modulate *KRAS* signaling

As shown in the two above-mentioned studies, as well as many others, many miRNAs have shown to target and regulate *KRAS* in cancer. However, the mechanisms by which those miRNAs are regulated may lead to a better understanding of cancer development and an opening of new therapeutic approaches. Two recent studies revealed how two *KRAS* targeting miRNAs are regulated transcriptionally and by other factors in the signaling pathway.

In a cohort of 224 human pancreatic neoplasms, Tanaka *et al*. reported a widespread overexpression of EVI1 oncogenic transcriptional factor in pancreatic ductal adenocarcinoma (PDAC) precursors and PDAC [10]. The group also uncovered that EVI1 functioned in proliferation and migration in pancreatic cancer cells and can modulate KRAS protein levels and KRAS-ERK pathway by transcriptionally regulating miR-96 and miR-181. Ectopic introduction of miR-96, but not miR-181, decreased KRAS protein expression and resulted in cell cycle arrest in cells, suggesting miR-96 as a tumor suppressor in EVI1-mediated *KRAS* regulation [10].

Using miRNA microarrays, Zhang *et al*. found that the MET receptor tyrosine kinase regulated miR-134 in glioblastoma (GBM) cells and glioblastoma stem cells (GSCs) [11]. Additionally, while miR-134 was down-regulated, multiple receptor tyrosine kinases (RTKs), MET, EGFR and PDGFR, were activated in GBM cells, GSCs and human tumors. The tumor-suppressive property of miR-134 was confirmed when overexpression of miR-134 inhibited proliferation in GBM cells and tumor growth in GSC-derived xenografts by targeting *KRAS* and *STAT5B*. MiR-134 regulation by RTK was mediated by MAPK and KLF4 transcription factor [11].

### Sequence variants as potential effectors in miRNA-mediated regulation of *KRAS*

In addition to dysregulation of miRNAs, sequence variants in the 3′ UTR of target mRNAs can affect their gene regulation. By sequencing the regions of the 3′ UTR of *KRAS* in multiple non-small cell lung cancer (NSCLC) cases, rs61764370 (also known as the *KRAS*-variant) was identified as the first single nucleotide polymorphism (SNP) within a *let*-*7* complementary site to be a biomarker for NSCLC risk [12]. The *KRAS*-variant has shown to function as a biomarker for risk of certain cancer types [13-16] and endometriosis [17], as well as a predictor for drug response [18,19]. However, the universality of this marker remains to be further investigated as some studies failed to show an association between the *KRAS*-variant and cancer risk [20,21] and drug response [22]. In addition, two recent studies on the patients enrolled in clinical trials found no association between stage 3 colon cancer and the variant in a large cohort [23], and no significant association between endometrial cancer and the variant due to a limited sample size [24].

Additional sequence variants in the 3′ UTR of *KRAS* have been actively searched for and tested for their potential as biomarkers. While not as extensively validated as the *KRAS*-variant, another SNP in the 3′ UTR of *KRAS*, the rs712 variant, is being assayed as a biomarker for risk of oral squamous cell carcinoma, gastric, colorectal and papillary thyroid cancer [25-28]. Although no novel NSCLC-associated variant was identified from a recent effort by Kim *et al*. due to a small sample size [29], this study, as well as a study by Sabarinathan *et al*. [30] suggested that some SNPs can disrupt proper miRNA-mediated *KRAS* regulation by destroying miRNA complementary sites and changing the secondary structures of the RNA.

### Conclusions and future directions

Understanding the molecular mechanism of miRNA-mediated regulation of *KRAS* by characterizing tumor suppressive miRNAs and oncoproteins that regulate tumor suppressive miRNAs in the *KRAS* signaling pathway would be beneficial for developing treatments in the clinic. In addition, the discovery of a validated sequence variant as a cancer biomarker for prognosis, diagnosis and treatment response would provide a valuable clinical tool. For example, many studies are examining the potential of the *KRAS*-variant as a cancer biomarker. However, the universal clinical relevance of the *KRAS*-variant remains unclear. Since cancer is a very heterogeneous disease, and many confounding factors such as population, age and external factors can affect the outcome, rigorous case-control studies are warranted to confirm the clinical application of variants as biomarkers for specific cancers.
